# 21 Decreasing Preparation Time of Full-thickness Engineered Constructs: Seeding Dermal Allografts with Non-cultured Keratinocytes

**DOI:** 10.1093/jbcr/irac012.025

**Published:** 2022-03-23

**Authors:** Summer Gallentine, Heather Powell

**Affiliations:** The Ohio State University, Columbus, Ohio; The Ohio State University, Columbus, Ohio

## Abstract

**Introduction:**

Early wound closure reduces risk of infection and fluid loss, which can reduce mortality and decrease length of hospital stay. The most common challenge associated with rapid wound closure in patients with large, full-thickness (FT) burns is lack of available donor skin. Tissue engineering can generate significant amounts of tissue for grafting but requires specialized facilities and up to three weeks of culture, substantially increasing time to closure.

Off-the-shelf technologies that do not require *ex vivo* culture of cells represent a major advance in wound care. Point-of-care cell spray technology isolated using an autologous cell harvesting device (ACHD) allows for immediate application of a cell suspension to the wound bed, eliminating specialized laboratory equipment and culture of skin cells. The goal of this project was to assess the ability of freshly isolated skin cells harvested using ACHD or traditional laboratory techniques to regenerate epithelium on engineered dermal tissue, in an effort to decrease time required for preparation of FT engineered skin.

**Methods:**

Dermal allografts were made by seeding collagen scaffolds with human dermal fibroblasts. Human surgical discard tissue was collected with IRB approval. Split-thickness skin (0.008”) was harvested from the discard tissue and cells were isolated using ACHD or via standard laboratory procedures following epidermal separation. Cell suspensions were seeded on the dermal allografts and cell populations were assessed histologically using H&E and Fontana-Masson’s, and via immunohistochemistry for Ki67, ColIV, ColVII, keratin 15, and involucrin. Advanced phenotyping of the cell suspension isolated using ACHD was assessed using flow cytometry.

**Results:**

Isolation of cells using ACHD required 8-fold less preparation time and resulted in a different ratio of cell constituents. ACHD yielded suspensions comprised predominantly of keratinocytes (60-75%), fibroblasts (25-40%), and melanocytes (1-3%). Advanced phenotyping indicates subpopulations of keratinocytes including epidermal stem cells, activated keratinocytes, and proliferating keratinocytes. The standard laboratory protocol resulted in a keratinocyte suspension (96-99%) with melanocytes (1-3%) and no fibroblasts. Both cell suspensions were capable of epidermal regeneration with greater pigment and slightly higher numbers of proliferative cells and epidermal stem cells with the ACHD.

**Conclusions:**

The results suggest non-cultured cell sprays have the potential to regenerate bioengineered dermal allografts in the absence of a split-thickness skin graft. Additionally, ACHD offers a significant time advantage to laboratory-based procedures in the preparation of cell-spray.

